# Formation Dominates Resorption With Increasing Mineralized Density and Time Postfracture in Cortical but Not Trabecular Bone: A Longitudinal HRpQCT Imaging Study in the Distal Radius

**DOI:** 10.1002/jbm4.10493

**Published:** 2021-04-08

**Authors:** Penny R Atkins, Kerstin Stock, Nicholas Ohs, Caitlyn J Collins, Lukas Horling, Stefan Benedikt, Gerald Degenhart, Kurt Lippuner, Michael Blauth, Patrik Christen, Ralph Müller

**Affiliations:** ^1^ Institute for Biomechanics, ETH Zurich Zurich Switzerland; ^2^ Department of Osteoporosis Bern University Hospital, University of Bern Bern Switzerland; ^3^ Department of Orthopedics and Trauma Surgery Medical University of Innsbruck Innsbruck Austria; ^4^ Department of Radiology Medical University Innsbruck Innsbruck Austria; ^5^ Clinical Medical Department DePuy Synthes Zuchwil Switzerland; ^6^ Institute for Information Systems FHNW University of Applied Sciences and Arts Northwestern Switzerland Olten Switzerland

**Keywords:** ANALYSIS/QUANTITATION OF BONE, BONE QUANTITATIVE COMPUTED TOMOGRAPHY (QCT), BONE MICRO‐COMPUTED TOMOGRAPHY (μCT), INJURY/FRACTURE HEALING, ORTHOPAEDICS, RADIOLOGY

## Abstract

Clinical evaluation of fracture healing is often limited to an assessment of fracture bridging from radiographic images, without consideration for other aspects of bone quality. However, recent advances in HRpQCT offer methods to accurately monitor microstructural bone remodeling throughout the healing process. In this study, local bone formation and resorption were investigated during the first year post fracture in both the fractured (n = 22) and contralateral (n = 19) radii of 34 conservatively treated patients (24 female, 10 male) who presented with a unilateral radius fracture at the Innsbruck University Hospital, Austria. HRpQCT images and clinical metrics were acquired at six time points for each patient. The standard HRpQCT image acquisition was captured for all radii, with additional distal and proximal image acquisitions for the fractured radii. Measured radial bone densities were isolated with a voxel‐based mask and images were rigidly registered to images from the previous imaging session using a pyramid‐based approach. From the registered images, bone formation and resorption volume fractions were quantified for multiple density‐based thresholds and compared between the fractured and contralateral radius and relative to demographics, bone morphometrics, and fracture metrics using regression. Compared with the contralateral radius, both bone formation and resorption were significantly increased in the fractured radius throughout the study for nearly all evaluated thresholds. Higher density cortical bone formation continually increased throughout the duration of the study and was significantly greater than resorption during late‐stage healing in both the fractured and intact regions of the radius. With the small and diverse study population, only weak relationships between fracture remodeling and patient‐specific parameters were unveiled. However this study provides methods for the analysis of local bone remodeling during fracture healing and highlights relevant considerations for future studies, specifically that remodeling postfracture is likely to continue beyond 12‐months postfracture. © 2021 The Authors. *JBMR Plus* published by Wiley Periodicals LLC on behalf of American Society for Bone and Mineral Research.

## Introduction

Distal radius fractures are the most common fracture in adults and, when resultant of low‐energy trauma, are indicative of possibly underlying osteoporosis and decreased bone quality.^(^ ^1^, ^2^, ^3^ ^)^ The clinical assessment of healing in these fractures often includes patient‐reported pain and function combined with radiographic assessment of cortical bridging. Although measures of pain and function are subjective and cannot provide direct insight to fracture union,^(^ ^4^ ^)^ radiographic assessment provides visualization of cortical bridging that directly indicates healing. Unfortunately, the lack of standardization and the absence of density‐based calibration prevents the use of radiographic images to observe bone quality through the process of fracture healing, which may be relevant given the increased prevalence of these fractures in older patients with increased risk of osteoporosis or other bone metabolic diseases affecting cortical and/or trabecular bone compartments.

Three‐dimensional (3D) imaging with calibrated CT provides insight into both bone density and structure; however, the adoption of CT imaging in the assessment of fracture healing has been limited, likely because of the increased radiation in comparison with radiographs and the difficulty in assessing cortical bridging caused by low image resolution. Recently, HRpQCT, which boasts both lower radiation and higher resolution than clinical CT, has been adopted to observe microstructural and morphological changes throughout both early‐ and late‐stage postfracture fracture healing.^(^ ^5^, ^6^, ^7^ ^)^ HRpQCT imaging has provided the data necessary to observe changes in both bone density and microarchitecture during the first 3‐months postfracture and to confirm that these aspects of bone health resemble those of the contralateral radius by 2‐years postfracture.^(^ ^6^, ^7^ ^)^ Importantly, these analyses of density and microarchitecture may provide insight into the healing process and are part of the standard patient evaluation built‐in to the scanner software. However, recent studies have shown that preprocessing of the images using rigid 3D registration provides a more accurate method to assess changes in bone microarchitecture because the same region of interest (ROI) can be analyzed in longitudinally acquired images.^(^ ^8^ ^)^ Longitudinal imaging and 3D‐image registration are tools provided in the scanner software, but have yet to be adopted into the standard patient evaluation for HRpQCT images. Although these techniques have allowed for quantification of localized microstructural remodeling in cohorts of 10 or fewer patients with decreased bone quality and disease,^(^ ^9^, ^10^, ^11^ ^)^ the methods used to date in patients have diverged from the methods used to quantify formation and resorption in animal studies^(^ ^12^, ^13^ ^)^ and have been inconsistent in the specific parameters used for quantification of remodeling (i.e., mineral density‐based thresholds and noise reduction techniques). However, concurrent research has also shown that 3D‐image registration can be applied to fractured radii and even help to monitor changes in BMD in localized regions of the fracture.^(^ ^14^, ^15^ ^)^ Importantly though, these studies have been limited to proof of concept because of their evaluation of only small ROIs and three or four patients, respectively. Thus, by applying the combined techniques of 3D‐image registration and quantification of bone formation and resorption to images of healing fractured radii, we may be able to specifically and locally quantify microstructural changes in BMD and structure throughout the duration of fracture healing.

The purpose of this study was to develop and apply an analysis protocol similar to that used in animal studies aimed at facilitating the use of time‐lapse HRpQCT imaging of the distal radius to investigate local bone formation and resorption during fracture healing, both in the fracture region and in the surrounding intact bone. We hypothesized that through use of this analysis protocol in combination with results from the contralateral radius, we would observe increased formation followed by resorption in the fracture region and that remodeling during fracture healing would be influenced by bone quantity and quality, patient demographics, and clinical outcome measures.

## Patients and Methods

### Participants

A subset of 34 patients of the 106 recruited for a time‐lapse HRpQCT imaging study were included herein based on the availability of high‐quality data from imaging sessions (Table 1). All patients provided informed consent before participation in our study approved by the Ethics Committee of the Medical University of Innsbruck (UN 0374344/4.31). Inclusion criteria were age of 18 years, unilateral distal radius fracture treated conservatively with a plaster cast for 5 weeks, and the absence of other conditions expected to affect the HRpQCT measurements (e.g., conditions restricting hand movement or affecting bone metabolism, ongoing treatment with steroids, radiation, or chemotherapy). Patient demographics including age, sex, BMI, and arm dominance in relation to the fracture were recorded.

**Table 1:** Demographic and Morphometric Measures of Study Subgroups

	Metric	Fracture subgroup	Contralateral subgroup	*p* Value
Demographics and descriptors	Sex	17 Female, 5 Male	13 Female, 6 Male	0.725
	Radius fractured^a^	7 Dominant 3 Ambidextrous 12 Nondominant	9 Dominant 2 Ambidextrous 8 Nondominant	0.620
	Fracture mechanism^a^	16 Low impact 6 High impact	11 Low impact 8 High impact	0.215
	Age, y^b^	55 ± 17	50 ± 17	0.357
	BMI, kg/m^2^ ^b^	23.6 ± 3.6	23.6 ± 3.3	0.992
Morphometric measures of the contralateral radius	Tb.BV/TV^b^	0.173 (0.041)	0.192 (0.059)	0.058
	Tb.Th, mm^c^	0.216 (0.024)	0.223 (0.025)	0.216
	Tb.Sp, mm^c^	0.777 (0.168)	0.734 (0.127)	0.066
	Tb.N, mm^−1^ ^b^	1.214 ± 0.189	1.287 ± 0.195	0.241
	Tb.vBMD, mg HA/cm^3^ ^c^	120.0 (36.0)	133.4 (42.3)	0.042
	Ct.Th, mm^b^	0.800 ± 0.243	0.876 ± 0.355	0.096
	Ct.Po, %^c^	0.007 (0.010)	0.006 (0.005)	0.309
	Ct.vBMD, mg HA/cm^3^ ^c^	824.0 (154.0)	861.5 (109.3)	0.079
	Tt.vBMD, mg HA/cm^3^ ^b^	232.0 ± 62.7	265.1 ± 70.5	0.129
DXA *T* score	Radius^b^	−1.8 ± 1.2	−1.3 ± 1.3	0.215
	Femur^b^	−0.9 ± 0.9	−0.7 ± 0.9	0.426
	Spine^b^	−1.5 ± 1.1	−0.9 ± 1.0	0.121

Abbreviations: BV/TV, bone volume fraction; HA, hydroxyapatite; N, number; Po, porosity; Sp, separation; Tb, trabecular; Th, thickness; vBMD, volumetric bone mineral density. Note: All morphometrics reported for the contralateral (intact) radius. Comparisons between participants with fractures and contralateral radii were not corrected for multiple comparisons.

^a^ Count; Fisher's exact test.

^b^ Mean ± SD; Student's *t* test.

^c^ Median (interquartile range); Mann–Whitney *U* test.

### Image and clinical data acquisition

**Fig 1:**
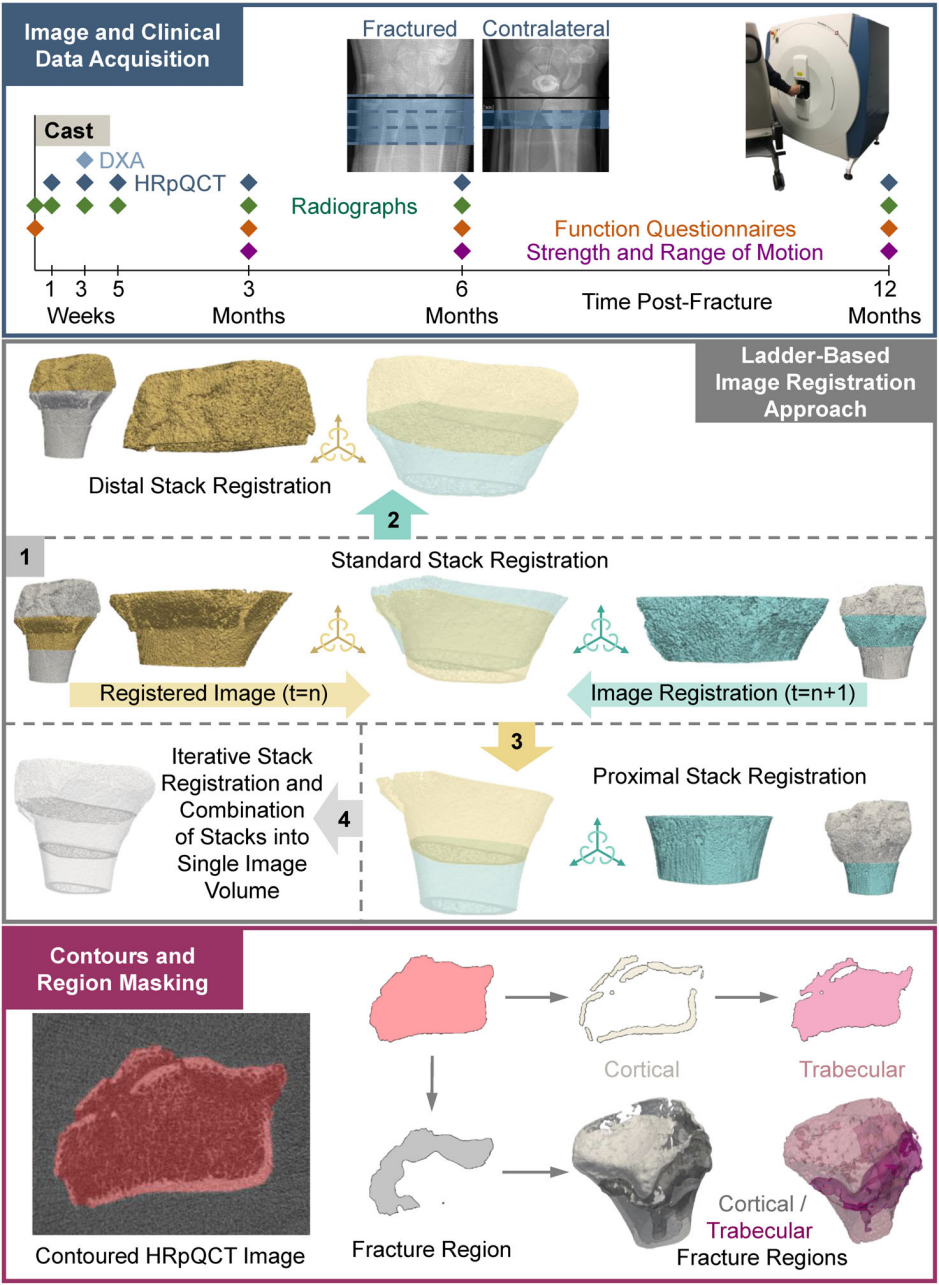
Data collection and processing overview. HRpQCT images were acquired of both the fractured and contralateral radius and ulna at six time points over the first‐year postfracture along with DXA and radiographic images and functional assessments, including questionnaires and measures of strength and range of motion at specific time points (*top*). HRpQCT images were registered using a rigid registration approach for the contralateral and middle stack of the fractured radius (*middle*). The registration of the proximal and distal imaging stacks of the fractured radius used a ladder‐based approach that leveraged differences in placement of the reference line during scanning to align the three imaging stacks of each bone that were then combined into a single imaging volume. Registered images were masked to isolate the radius using the methods of Ohs and colleagues, then divided into cortical and trabecular regions using a threshold‐based approach and into fracture and intact regions from manual identification of the fracture (*bottom*).

HRpQCT (XtremeCT II; Scanco Medical AG) images were acquired 1‐week, 3‐weeks, 5‐weeks, 3‐months, 6‐months, and 12‐months postfracture (60.7‐μm isotropic voxels, 63 kV, 1500 μA, 46‐ms integration time, 2304 samples, 900 projections). At each imaging session, three adjacent, nonoverlapping image acquisitions or stacks (220–504 slices, median = 500 slices, corresponding to a size of the ROI of 13.4–30.6 mm, median = 30.4 mm where image stacks ranged from 68 to 168 slices in length) were acquired for the fractured arm and one standard image stack (168 slices, 10.2 mm) was acquired for the contralateral arm. The effective radiation dose of all acquired HRpQCT images was 0.837 to 0.909 mSv per study participant, which is far below the 50 mSv yearly limit for radiation workers. All images were acquired relative to a reference line placed at the inflection between the articulating surface of the radius with the scaphoid and lunate (Fig. 1). Imaging of the fractured arm began 1.2‐mm distal to the reference line, whereas imaging of the contralateral arm began 9.0 mm proximal to the reference line. With this protocol, the reference line could be placed at the same location for both the contralateral and fractured radii and the middle image stack of the fractured arm was approximately aligned with the standard image stack of the contralateral arm when the three image stacks consisted of 168 slices; however, there was some variability in placement of the reference line to allow full imaging of the fracture. Additionally, the imaging protocol had originally allowed for specification of the length of each image stack, which resulted in the large deviations in stack length. Nevertheless, a majority of images of the fractured radius consisted of 167 or 168 slices per stack.

Image quality was assessed relative to the visual grading score (VGS), which ranges on a scale from 1 (no motion artifact) to 5 (major streaking, trabecular smearing, and cortical disruptions) by one researcher and one clinician.^(^ ^16^ ^)^ All scores agreed within one grading level. In cases where there was a discrepancy, the worse score was conservatively recorded (e.g., for scores of VGS 3 and VGS 2, the recorded score was VGS 3). Only patients with sufficient quality images (VGS 1–3) for all six time points of either the fractured or contralateral radius were included in this analysis, which excluded 72 of the total 106 patients recruited for the study and resulted in the inclusion of 22 fractured radii and 19 contralateral radii, with seven participants included in both the fracture and contralateral subgroups. Bone‐to‐soft tissue signal‐to‐noise ratio (BST‐SNR) was calculated using the mean mineralized density value within the cortical mask and the SD of the region immediately surrounding the radius for all registered images. Although this varies from the definition of SNR, where the SD of only background is used, it better represents the analyzed portion of the images because it highlights the contrast between bone tissue and its environment consisting of mineralizing callus and soft tissue.

DXA images of the lumbar spine, proximal femur, and contralateral radius (if not previously fractured: one patient in the fracture subgroup had a prior fracture of the contralateral radius, no patients of the contralateral subgroup had a prior fracture) were acquired 3‐weeks postfracture and T scores were recorded. Standard distal radiographic images of the fractured forearm were acquired at the time of fracture, as well as at each imaging session. Dorsal‐palmar inclination, ulnar variance, and radial inclination were measured on each radiograph. Fractures were classified using the Orthopedic Trauma Association (OTA) fracture and dislocation classification,^(17^ ^)^ and the injury mechanism was classified as either a high‐ or low‐impact fall. Patient function was measured using the Disabilities of the Arm, Shoulder, and Hand (DASH), Patient‐Rated Wrist Evaluation (PRWE), and the Michigan Hand Questionnaire (MHQ) grading systems. Patients were asked to report baseline function before fracture and then completed questionnaires again at the 3‐, 6‐, and 12‐month imaging sessions. Range of motion (ROM) in all three planes of motion and grip strength was measured at the 3‐, 6‐, and 12‐month imaging sessions and used to assess fracture healing.

### Image registration and region masking

The region of the radius of each HRpQCT image was isolated and registered to the radius of an adjacent image using a pyramid‐based approach^(^ ^18^ ^)^ which iteratively aligned adjacent images to allow for direct comparisons of BMD (Fig. 1, Supplementary Information S1). The separate stacks of the fractured radius were registered using a ladder‐based approach between images of adjacent time points. The radius of each registered image was then contoured using a geodesic active contouring method.^(^ ^19^ ^)^ Within this contour, both cortical and trabecular regions were isolated, as well as the region of the fracture (Supplementary Information S1).

### Densitometry and static morphometry

Standard patient morphometrics were quantified from an unregistered image stack of the contralateral radius using image processing language (IPL), which is part of the scanner manufacturer software. Densitometric indices, including volumetric BMD (vBMD) for the whole bone (Tt.vBMD), trabecular (Tb.vBMD), and cortical (Ct.vBMD) regions, and morphometric indices, including bone volume fraction (BV/TV), cortical porosity (Ct.Po), trabecular number (Tb.N), trabecular separation (Tb.Sp), and mean thickness of the trabecular (Tb.Th) and cortical (Ct.Th) regions, were evaluated to quantify bone quantity and quality of each study participant and compare subgroups of patients. Without available validation of commonly reported densitometric and morphometric measures on fractured and healing bone, only a single image stack of the contralateral arm was used for measurement of densitometry and static morphometry of each study participant. Further, since high quality contralateral images (VGS 1 through VGS 3) were not available at all time points for the fracture subgroup, a single image stack was used for consistency across all participants whether included in the fracture or contralateral subgroup. The evaluated contralateral image was chosen based on image quality, where higher quality was preferred based on known errors resultant of motion artifact.^(^ ^20^ ^)^ However, when multiple images had equivalently good quality, the latest of these images was chosen to minimize any effects on the measurement of these metrics based on systemic inflammation resultant from the fracture.

### Dynamic morphometry

Although bone formation rate and bone resorption rate have been the standards in reporting dynamic morphometry in animal studies,^(^ ^12^, ^21^, ^22^ ^)^ the investigation of remodeling in humans has instead used formation and resorption bone‐volume fractions, which do not include the aspect of time between evaluated images.^(^ ^9^, ^10^, ^11^ ^)^ Although the specific reasoning behind this decision is unknown, the increased image noise of lower resolution clinical images from HRpQCT, in comparison with μCT images, would result in greater false‐positive and false‐negative remodeling events. Thus, the calculation of rates of change, such as bone formation rate and bone resorption rate, would be biased to the time interval between measurements. For this reason, in conjunction with the varied time intervals of our study, we have reported remodeling in terms of formation and resorption volume fractions.

Before the calculation of formation and resorption volume fractions, registered density images were filtered with a constrained Gaussian filter (σ = 0.8, truncate = 1.25, support = 1.0). Thresholds of densities between 200 and 960 mg hyaluronic acid (HA)/cm^3^ with an interval of 120 mg HA/cm^3^ were applied to the Gaussian‐filtered images. Voxels at each density threshold were summed for the central slice of each analyzed image stack to assess the distribution of bone density in the various ROIs. Bone formation and resorption volume fractions were then calculated for each density as the volume of bone formed or resorbed divided by the total volume of bone of that density at the earlier imaging session.^(^ ^12^, ^13^ ^)^ All formation and resorption calculations were completed relative to the following time point, such that formation and resorption bone‐volume fractions presented for 1‐week postfracture are relative to 3‐weeks postfracture and those for 3‐weeks postfracture are relative to 5‐weeks postfracture. At any given density threshold, formation voxels were those in the latter image, but not the former, whereas resorption voxels were in the former image, but not the latter image. The cortical region of evaluation was additive between each set of two time points evaluated such that any voxel labeled as cortex in either image would be considered cortex. The trabecular mask then included all noncortical voxels within the set of periosteal contours. All of the density thresholds were evaluated for the cortical region, whereas only densities ranging from 200 to 680 mg HA/cm^3^ were evaluated for the trabecular region, based on the lower mineralized density of trabecular bone and minimal trabecular bone volume measured at higher densities.

### Statistical analysis

Using the Python SciPy function library, results were evaluated using the Shapiro–Wilk test of normality and then evaluated using the appropriate analysis for non‐normally or normally distributed results.^(^ ^23^ ^)^ Non‐normally distributed results were presented as median (interquartile range) and compared using Spearman's Rank correlation coefficient and a Wilcoxon or Mann–Whitney *U* test, as appropriate, whereas normally distributed results were presented as mean ± SD and compared using Pearson's correlation coefficient and a paired or unpaired Student's *t* test, as appropriate. Statistical analyses that compared the fracture and contralateral data accounted for the potential dependence between the fracture and contralateral radii of the seven participants included in both subgroups with the use of a partially paired analysis, as described by Derrick and colleagues, which consider both the paired and independent samples without an introduction of bias.^(^ ^24^ ^)^ The ANOVA was used to determine group differences relative to multiple factors, where the Kruskal‐Wallis *H* test was used to evaluate group differences for nonparametric results. All comparisons of formation and resorption were calculated on the magnitude of the volume fraction. Corrections for multiple comparisons were applied to results that compared metrics of healing (i.e., formation, resorption, strength, ROM, or function) using the Python Statsmodels function library implementation of the Holm‐Bonferroni correction.^(^ ^25^ ^)^ Differences in BST‐SNR were evaluated across appointments and between groups using a mixed linear regression model in the Python Statsmodels function library to determine relevant factors that affected BST‐SNR.^(^ ^25^ ^)^

Repeated‐measures ANOVA was used to identify whether time frame, bone type (trabecular or cortical), density threshold, direction of remodeling (formation or resorption), and region (fracture or intact) affected the magnitude of the formation or resorption volume fractions for both the contralateral and fractured radius. To further investigate the specific parameters that had the largest effect on formation and resorption, partial least squares (PLS) regression was performed on each formation and resorption volume fraction including variables of demographics, morphometric data, injury descriptors (fracture side relative to hand dominance, low/high injury impact, AO/OTA fracture classification^(^ ^17^ ^)^), femur and lumbar spine DXA‐measured *T* scores,^(^ ^26^ ^)^ observed differences in radiographic measures, prescribed medication (vitamin D, calcium, and/or antiresorptive therapy), ipsilateral formation and resorption volume fractions, and density threshold using the Python Scikit‐Learn function library.^(^ ^27^ ^)^ Thresholds used only for the cortical regions, ROM, grip strength, and radius *T* scores were not considered in the PLS regression model because of the difficulties of assessing incomplete data sets (i.e., those not measured at all imaging sessions). All variables were scaled and centered before analysis such that they had a center value of 0 and a SD of 1. Leave‐one‐out cross validation was used to calculate the predictive power of the model because of the small number of data points and large number of variables evaluated. The number of model components was determined based on the Q^2^ value of the cross validation, such that additional components were only included if significant improvements to the model occurred, which were identified by increases in Q^2^ of at least 0.0975.^(^ ^28^, ^29^ ^)^ The variables were sorted by variable influence on projection and the model was run iteratively, including additional variables until the Q^2^ score no longer improved.

## Results

From the 34 patients with sufficient image quality who were included in this study, images from all six time points of the fractured radius were of sufficient quality for 22 patients and of the contralateral radius for 19 patients (both the fractured and contralateral radii images were of sufficient quality for seven patients). The two subgroups of patients evaluated did not differ in demographics or measures from densitometry, morphometry, or DXA, except for Tb.vBMD (*p* = 0.042; Table 1), and participant morphometrics were within recently published value ranges.^(^ ^30^ ^)^ All patients had good fracture healing outcomes without radiographic evidence of delayed or nonunion. Function before fracture was reported to be 0.00 (3.12) on the DASH and 0.0 (1.5) on the PRWE, both of which approached a score of 0, which indicates no disability. On the MHQ, function before fracture was reported to be 99.43 (6.77) for the contralateral and 99.36 (3.87) for the fractured arm that approached a score of 100, which indicates no disability for the MHQ. For the DASH and PRWE, these baseline measures were used for comparison of the scores at later time points, whereas for the MHQ, the contralateral arm scores were used, which showed no significant difference compared with the fractured arm before fracture (*p* = 0.952 at baseline). Questionnaire results, measures of strength, and ROM indicated functional recovery by 6‐months postfracture (Table 2), with many measures being equivalent by 3‐months postfracture.

**Table 2:** Functional, Strength, and Range of Motion Measures for Patients With Fracture During Late‐Stage Fracture Healing

		3‐Months postfracture		Six‐Months postfracture		12‐months postfracture	
		Median (IQR)	*p* Value	Median (IQR)	*p* Value	Median (IQR)	*p* Value
Function	DASH^a^	4.17 (11.04)	0.040	4.17 (9.17)	0.238	2.48 (7.89)	1.000
	PRWE^a^	7.5 (6.0)	0.022	3.3 (12.7)	0.305	0.0 (6.2)	1.000
	MHQ^b^	97.32 (7.97) 75.83 (9.15)	0.008	99.55 (5.48) 82.91 (20.21)	0.298	99.70 (2.45) 96.42 (17.15)	1.000
Strength (kg)^b^	Dominant	18.8 (8.2) 16.1 (4.6)	0.324	20.5 (6.9) 18.4 (9.2)	1.000	21.1 (9.7) 21.7 (8.4)	1.000
	Nondominant	25.0 (10.1) 17.8 (12.5)	0.009	23.4 (4.9) 21.8 (11.0)	0.776	25.4 (6.7) 21.5 (10.1)	0.376
	Ambidextrous	17.9 (8.2) 20.0 (6.9)	–	19.2 (6.9) 21.9 (7.6)	–	21.6 (9.7) 21.9 (7.8)	–
Range of motion (°)^b^	Flexion	59 (20) 45 (16)	0.008	58 (13) 54 (23)	0.520	60 (16) 56 (18)	0.933
	Extension	67 (17) 62 (16)	0.042	67 (16) 64 (20)	1.000	67 (13) 72 (15)	0.482
	Radial deviation	24 (10) 22 (10)	0.324	21 (8) 22 (10)	1.000	28 (16) 24 (20)	1.000
	Ulnar deviation	45 (14) 40 (9)	0.208	48 (13) 40 (13)	0.625	38 (22) 39 (16)	1.000
	Supination	81 (8) 78 (10)	0.208	84 (5) 85 (10)	1.000	85 (12) 85 (6)	1.000
	Pronation	84 (5) 84 (6)	0.324	83 (6) 83 (5)	1.000	85 (6) 85 (9)	1.000

Abbreviations: DASH, disabilities of the arm, shoulder, and hand; IQR, interquartile range; MHQ, Michigan hand questionnaire; PRWE, patient‐rated wrist evaluation. Note: All *p* values were corrected across measures for each time point postfracture.

^a^ Comparison with measurement at baseline.

^b^ Comparison with contralateral measurement, data listed as contralateral (top) and fracture (bottom).

Of the 34 patients with fractured radii, 19 patients were recommended to start some form of medication or supplement in support of fracture healing. Recommendations were provided based on standard clinical practices, considering the fracture, DXA measures, and clinically analyzed blood samples of the patients. Generally, the two subgroups were prescribed treatment similarly; a total of nine patients began vitamin D therapy (3‐weeks to 12‐months postfracture), two began calcium therapy (5‐weeks postfracture), and eight began a combined vitamin D and calcium therapy (3‐ to 5‐weeks postfracture); one patient who was included in both the contralateral and fracture subgroups was taking vitamin D, calcium, and Actonel (antiresorptive therapy) throughout the study.

### HRpQCT image analysis

Patients from both the fracture and contralateral radius subgroups showed no differences in timing of follow‐up appointments (Table 3). The fracture cohort did have better image quality in comparison with the contralateral radius group at all imaging sessions except 3‐ and 6‐months postfracture, which was consistent with our observation for all study participants and the reason why fewer contralateral than fracture radii could be included in this analysis.

**Table 3:** Description of Imaging Session‐Timing Postfracture and Image Quality

	Metric	Fracture subgroup			Contralateral subgroup			*p* Value
Postfracture time^a^	Appointment 1, d	8 (2)			8 (2)			0.430
	Appointment 2, d	22 (3)			23 (2)			0.267
	Appointment 3, d	37 (3)			37 (4)			0.468
	Appointment 4, d	87 (6)			86 (6)			0.255
	Appointment 5, d	172 (12)			176 (16)			0.248
	Appointment 6, d	356 (17)			358 (21)			0.490
Image quality^b^	Appointment 1	1:44	2:21	3:1	1:1	2:12	3:6	<0.001
	Appointment 2	1:43	2:17	3:6	1:1	2:14	3:4	<0.001
	Appointment 3	1:25	2:34	3:7	1:3	2:10	3:6	0.047
	Appointment 4	1:23	2:33	3:10	1:5	2:6	3:8	0.057
	Appointment 5	1:24	2:31	3:11	1:4	2:12	3:3	0.467
	Appointment 6	1:31	2:27	3:8	1:2	2:11	3:6	0.006

Note: Comparisons were not corrected for multiple comparisons.

^a^ Median (IQR); Mann–Whitney.

^b^ Count; Fisher's exact test.

**Fig 2:**
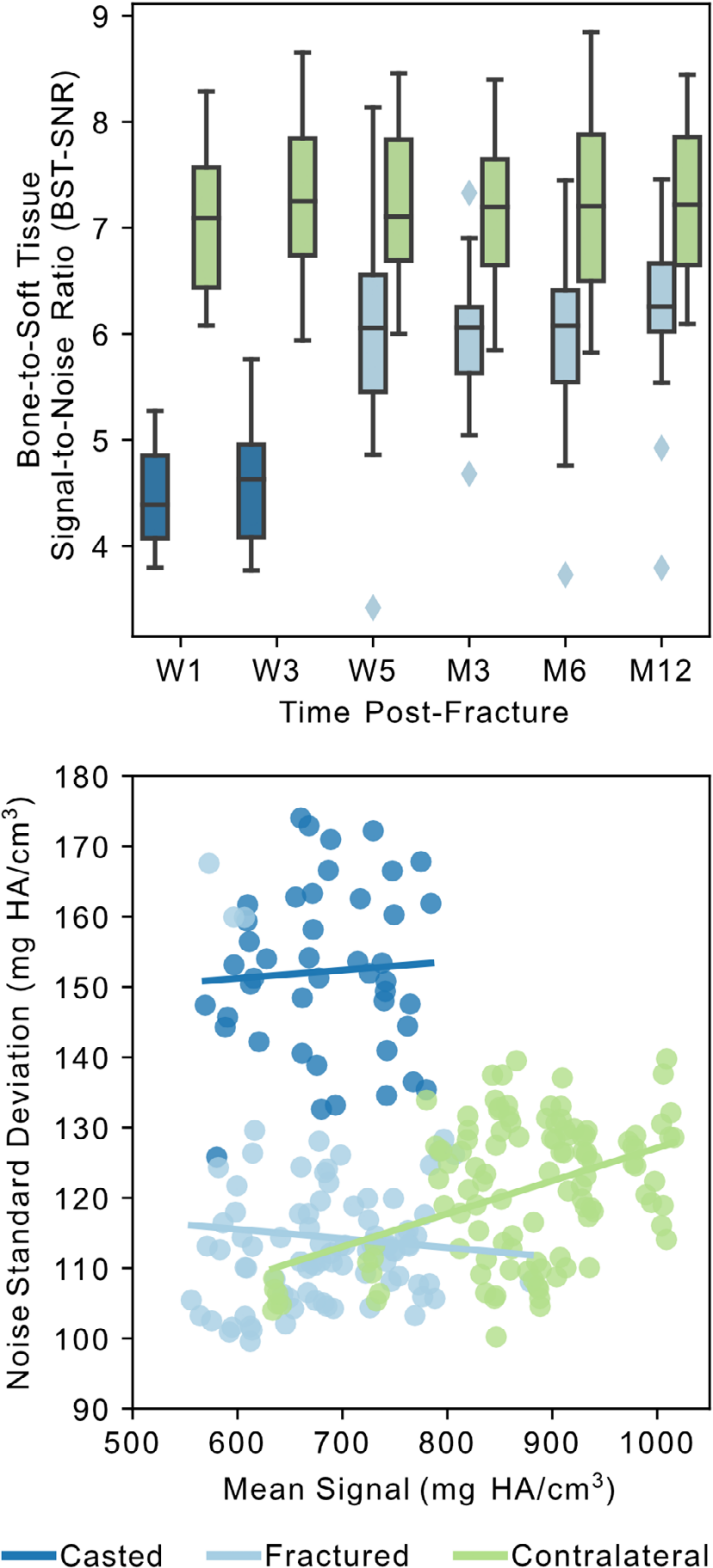
Analysis of image signal‐to‐noise ratio (SNR) for the three image categories: Casted = fractured radius with a cast; fractured = fractured radius without a cast; contralateral = contralateral radius. Bone‐to‐soft tissue‐ (BST‐)SNR was lowest for the casted images and highest for the contralateral images (*top*). SD of image noise in the region of soft tissue surrounding the radius versus mean image signal of cortical bone for the three image categories indicates a higher SD of noise for the casted images and a higher mean signal for the contralateral images (*bottom*). HA indicates hyaluronic acid; M, month; W, week.

The evaluation of BST‐SNR relative to three categories of images (fractured with cast, fractured without cast, and contralateral) and the six imaging sessions identified the three categories (*p* < 0.001 for each) and the 3‐week (*p* = 0.026) and 12‐month (*p* = 0.001) imaging sessions to be relevant in the prediction of BST‐SNR (Fig. 2). The images of casted fractured radii (1‐week and 3‐weeks postfracture) had decreased BST‐SNR caused by a larger SD of noise, whereas the images of the contralateral radii had increased BST‐SNR caused by a higher mean signal. Mean signal and the SD of the noise were positively correlated in the contralateral images (*r* = 0.315; *p* = 0.001), but not for the images of the casted or cast‐free fractured radius.

**Fig 3:**
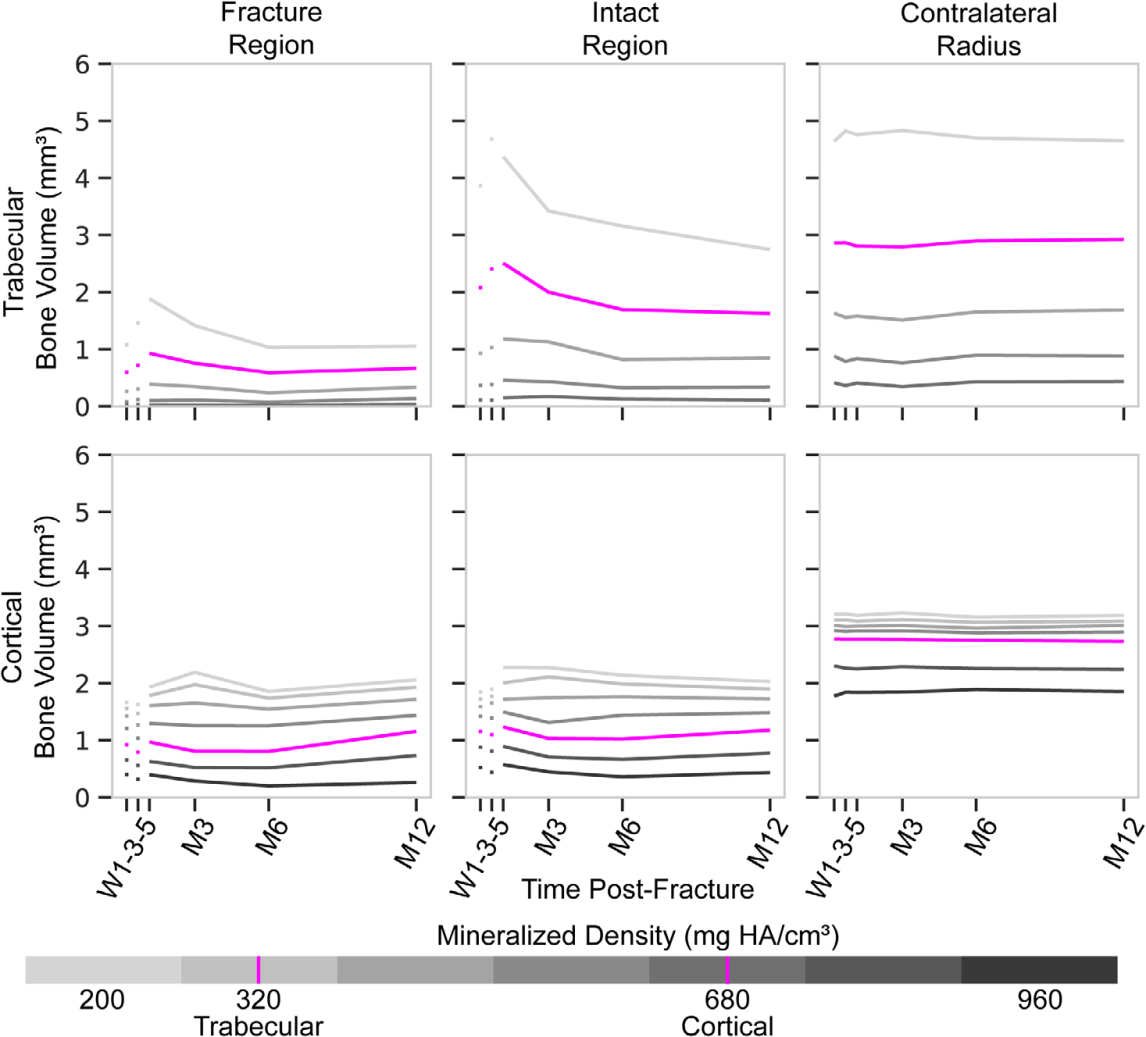
Summation of bone volume of a centrally located image for various density thresholds within each bone region. Density thresholds between 200 and 920 mg HA/cm^3^ were used for cortical regions. Density thresholds between 200 and 680 mg HA/cm^3^ were used for trabecular regions. Plots represent regions of the fractured (*left, center*) and contralateral radii (*right*). Solid line represents median value. HA indicates hyaluronic acid; M, month; W, week.

Bone volumes at each density threshold were consistent across the study for the contralateral radius (Fig. 3). For the fractured radius, overall volume of bone was approximately three times the volume of the contralateral images because of the increased stack length (median analyzed lengths of 22.00 mm for the fractured images compared with 6.92 mm for the contralateral images). However, there was large variability in the analyzed image length in both subgroups, which is why bone volumes were assessed for a single centrally located slice for all patients. Qualitatively, trabecular volumes at most densities peaked at 3‐weeks postfracture with the largest volumes and increases in volume observed for lower densities. Cortical volumes at each density decreased between 1‐week and 3‐weeks postfracture in both the fracture and intact regions. Cortical volumes increased between 3‐ and 5‐weeks postfracture for the fracture region, whereas for the intact region the change in cortical volume was density‐dependent (Fig. 3). Between 5‐weeks and 3‐months postfracture, lower density cortical volumes tended to increase, especially in the fracture region, whereas higher density volumes tended to decrease.

### Dynamic morphometry of the contralateral radius

**Fig 4:**
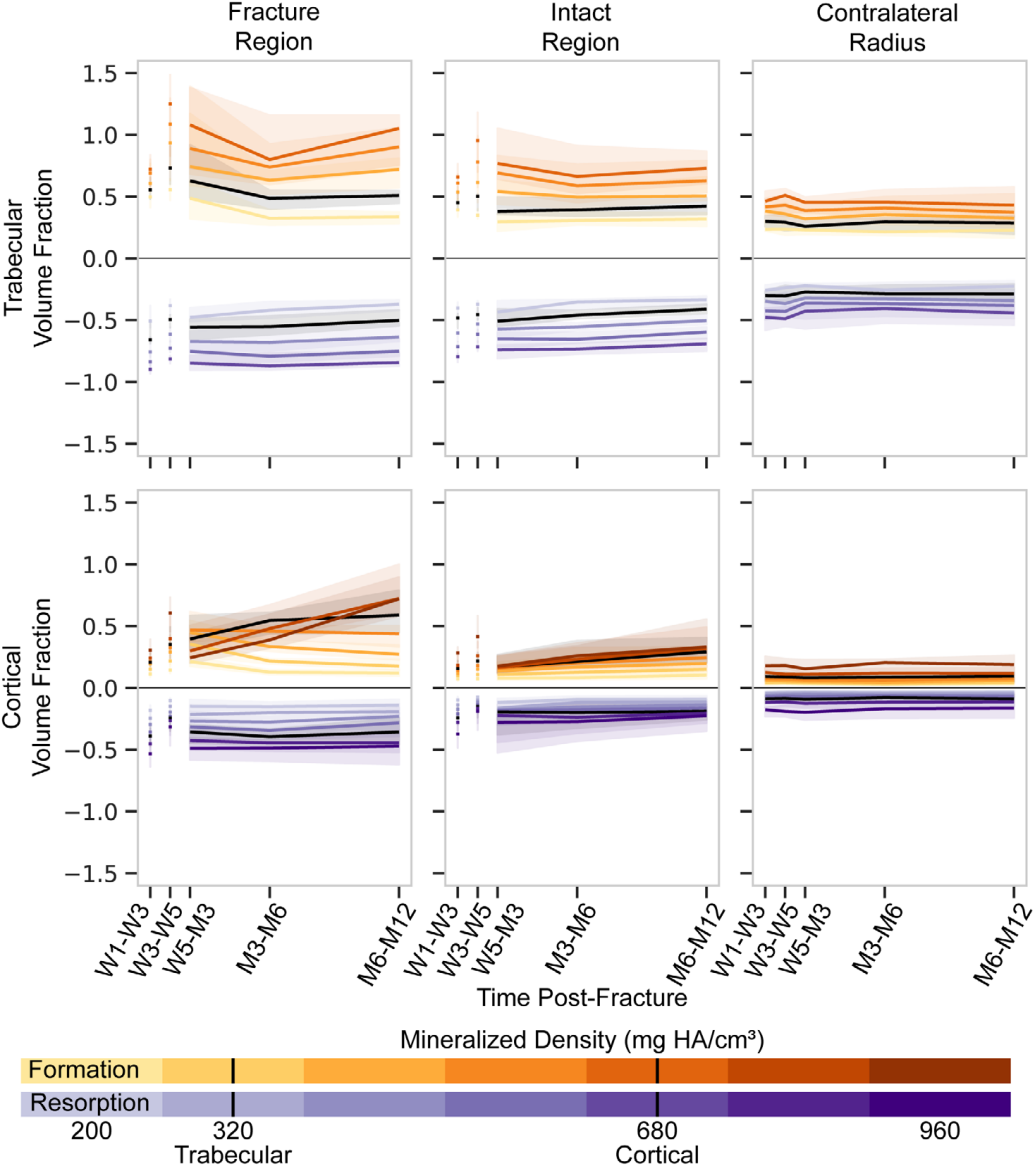
Longitudinal multidensity bone remodeling volume fractions for various thresholds within each region of bone. Thresholds between 200 and 920 mg HA/cm^3^ were used for cortical regions. Thresholds between 200 and 680 mg HA/cm^3^ were used for trabecular regions. Plots represent regions of the fractured (*left, center*) and contralateral radii (*right*). Solid line represents median value with a shaded interquartile range. HA indicates hyaluronic acid; M, month; W, week.

Quantification of the formation and resorption at multiple densities indicated consistent formation and resorption volume fractions in the contralateral radius over all time frames analyzed, even though time intervals ranged from 2 weeks to 6 months (Fig. 4). Bone type, density threshold, and the interaction between bone type and density threshold had an effect on the formation and resorption volume‐fraction magnitude for the contralateral radius, whereas the direction of the remodeling (formation or resorption) and the specific time frame did not (Table 4). Formation and resorption volume fractions in the trabecular and cortical regions of the contralateral radius were not independent of corresponding resorption and formation volume fractions, respectively (Table 5). However, only resorption in the trabecular region was predicted by remodeling outside of the specific bone region—resorption in the cortical region. For the thresholds of 320 and 680 mg HA/cm^3^ for trabecular and cortical bone, respectively, trabecular formation and resorption volume fractions were 0.294 (0.112) and 0.286 (0.121), respectively, whereas cortical formation and resorption volume fractions were 0.086 (0.070) and 0.087 (0.065), respectively, for the contralateral radius (Fig. 4). As these values did not change through the course of the study, this level of formation and resorption may instead represent the level of error in calculation caused by image quality, image noise, voxel size, thresholding, etc. Thus, these values were used as a reference for formation and resorption volume fractions of the fractured radius.

**Table 4:** Repeated Measures ANOVA Results for the Fracture and Contralateral Radius

Effect	Fracture				Contralateral			
	*df*1	F	*df*2	p Value	*df*1	F	*df*2	*p* Value
Appt	2	7.62	42	0.002	4	0.98	72	0.424
Tb‐Ct	1	588.37	21	0.000	1	406.05	18	0.000
F‐Rs	1	8.84	21	0.007	1	0.50	18	0.490
Fx‐Int	1	129.64	21	0.000				
Density	4	573.47	84	0.000	4	230.45	72	0.000
Appt:Tb‐Ct	2	3.46	42	0.041	4	1.49	72	0.214
Appt:F‐Rs	2	0.81	42	0.453	4	0.49	72	0.740
Tb‐Ct:F‐Rs	1	1.30	21	0.268	1	1.19	18	0.291
Appt:Fx‐Int	2	6.20	42	0.004				
Tb‐Ct:Fx‐Int	1	0.94	21	0.343				
F‐Rs:Fx‐Int	1	18.28	21	0.000				
Appt:Density	8	3.00	168	0.004	16	0.78	288	0.707
Tb‐Ct:Density	4	52.83	84	0.000	4	185.29	72	0.000
F‐Rs:Density	4	62.40	84	0.000	4	0.14	72	0.967
Fx‐Int:Density	4	65.80	84	0.000				
Appt:Tb‐Ct:F‐Rs	2	1.33	42	0.276	4	0.64	72	0.639
Appt:Tb‐Ct:Fx‐Int	2	2.38	42	0.105				
Appt:F‐Rs:Fx‐Int	2	7.39	42	0.002				
Tb‐Ct:F‐Rs:Fx‐Int	1	0.66	21	0.425				
Appt:Tb‐Ct:Density	8	5.22	168	0.000	16	0.86	288	0.611
Appt:F‐Rs:Density	8	1.44	168	0.184	16	0.38	288	0.987
Tb‐Ct:F‐Rs:Density	4	0.58	84	0.678	4	2.40	72	0.058
Appt:Fx‐Int:Density	8	5.79	168	0.000				
Tb‐Ct:Fx‐Int:Density	4	16.12	84	0.000				
F‐Rs:Fx‐Int:Density	4	16.92	84	0.000				
Appt:Tb‐Ct:F‐Rs:Fx‐Int	2	1.19	42	0.316				
Appt:Tb‐Ct:F‐Rs:Density	8	8.01	168	0.000	16	0.19	288	1.000
Appt:Tb‐Ct:Fx‐Int:Density	8	2.69	168	0.008				
Appt:F‐Rs:Fx‐Int:Density	8	5.13	168	0.000				
Tb‐Ct:F‐Rs:Fx‐Int:Density	4	6.40	84	0.000				
Appt:Tb‐Ct:F‐Rs:Fx‐Int:Density	8	1.18	168	0.312				

Appt, imaging session appointment number; Ct, cortical; F, formation; Fx, fracture; Int, intact; Rs, resorption; Tb, trabecular.

Note: Only uncasted data were included in this analysis: thus 3 “Appt” time points for the fractured radius and 5 for the contralateral radius.

**Table 5:** Results From Partial Least Squares Regression, Indicating the Significant Variable Coefficients and Importance on Projection for Each Remodeling Metric

		Fracture region			Intact region			Contralateral radius		
		Variable	VIP	Coef	Variable	VIP	Coef	Variable	VIP	Coef
Trabecular	Formation	Intercept Int Tb F Fx Ct F	‐ 1.12 0.86	0.544 0.226 0.116	Intercept Fx Tb F Density	‐ 1.03 0.97	−41.929 0.125 0.096	Intercept Tb Rs	‐ 1.00	0.316 0.120
		2 Components *R* ^2^ = 0.655, Q^2^ = 0.643			2 Components *R* ^2^ = 0.677, Q^2^ = 0.669			1 Component *R* ^2^ = 0.765, Q^2^ = 0.763		
	Resorption	Intercept Int Tb Rs	‐ 1.00	0.545 0.164	Intercept Fx Tb Rs	‐ 1.00	0.455 0.140	Intercept Tb F Ct Rs	‐ 1.10 0.89	0.330 0.070 0.057
		1 Component *R* ^2^ = 0.867, Q^2^ = 0.866			1 Component *R* ^2^ = 0.867, Q^2^ = 0.866			1 Component *R* ^2^ = 0.773, Q^2^ = 0.770		
Cortical	Formation	Intercept Int Ct F Fx Tb F Int Tb Rs	‐ 1.08 0.99 0.93	0.257 0.098 0.090 0.084	Intercept Int Ct Rs Fx Ct F	‐ 1.06 0.94	0.159 0.070 0.051	Intercept Ct Rs	‐ 1.00	0.065 0.042
		1 Component *R* ^2^ = 0.541, Q^2^ = 0.523			2 Components *R* ^2^ = 0.629, Q^2^ = 0.607			1 Component *R* ^2^ = 0.876, Q^2^ = 0.874		
	Resorption	Intercept Fx Tb Rs	‐ 1.00	0.204 0.099	Intercept Fx Ct Rs Int Ct F Int Tb Rs	‐ 1.03 1.03 0.94	0.147 0.033 0.043 0.016	Intercept Ct F	‐ 1.00	0.063 0.041
		1 Component *R* ^2^ = 0.617, Q^2^ = 0.612			2 Components *R* ^2^ = 0.668, Q^2^ = 0.659			1 Component *R* ^2^ = 0.876, Q^2^ = 0.874		

Abbreviations: BV/TV, bone volume fraction; Coef, regression coefficients; Ct, cortical; F, formation; Fx, fracture; Int, intact; Rs, resorption; Tb, trabecular; vBMD,volumetric bone mineral density; VIP, variable influence on projection.

Note: Only uncasted data was included in this analysis, thus only late‐stage fracture healing is evaluated for the fractured radius, whereas the entire year is included for the contralateral radius.

**Fig 5:**
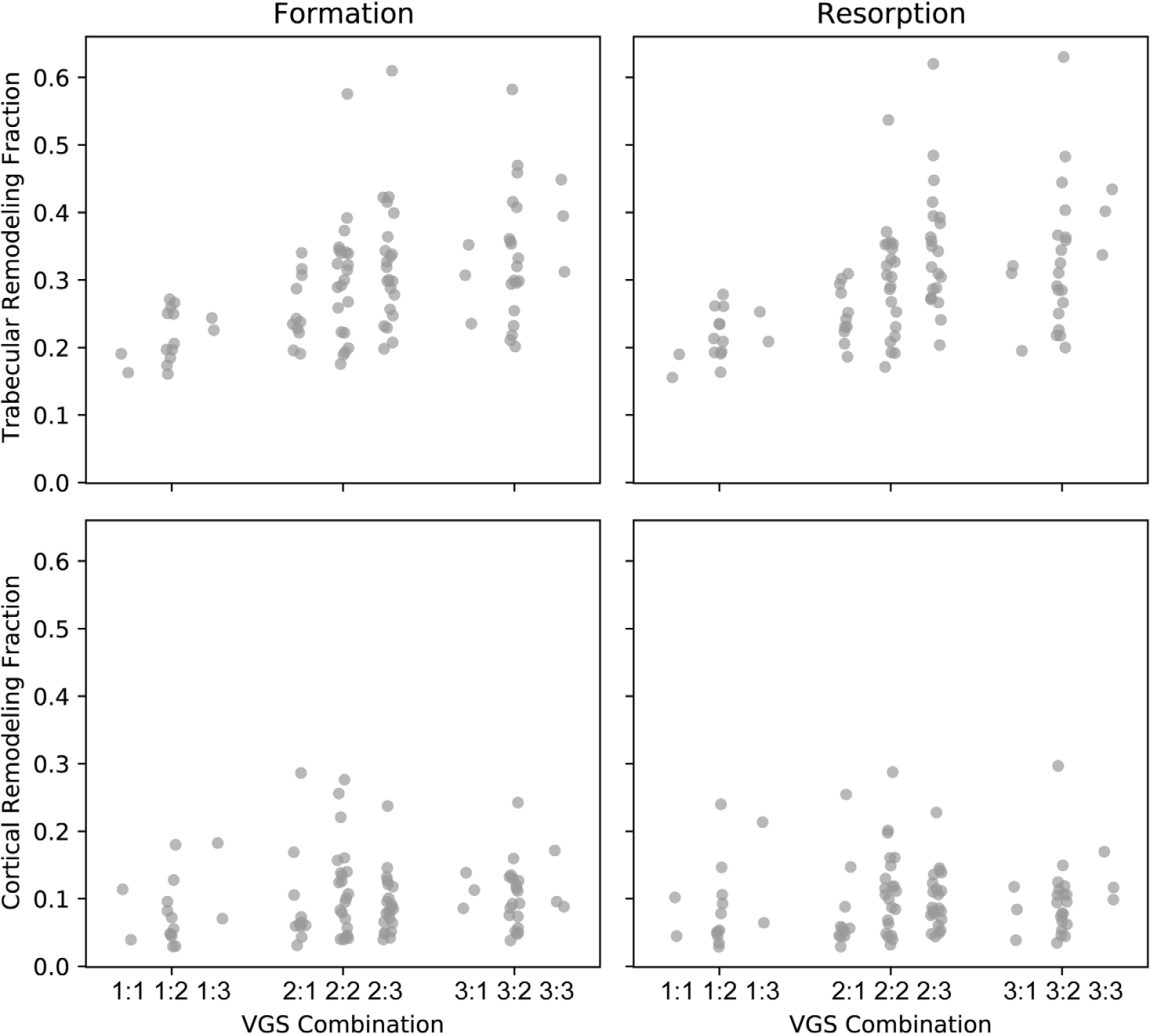
Reduced image quality, rated using the visual grading score (VGS), resulted in larger remodeling bone‐volume fractions in the contralateral images. Each VGS combination is listed as target:registered image.

Although generally only high‐quality images (VGS 1–3) were included herein, the image quality of both the target and registered image of each pair of registered images affected both formation and resorption volume fractions (*p* < 0.001 for the target image, *p* = 0.002 for the registered image; Fig. 5). Interestingly, when the formation and resorption volume fractions were compared for each image quality, VGS 1 images had lower formation and resorption rates than both VGS 2 and VGS 3 images as either a target (*p* > 0.001 for both VGS 2 and VGS 3) or a registered image (*p* = 0.031 for VGS 2 and *p* > 0.001 for VGS 3), but no differences were observed between VGS 2 and VGS 3.

### Dynamic morphometry of the fractured radius

**Fig 6:**
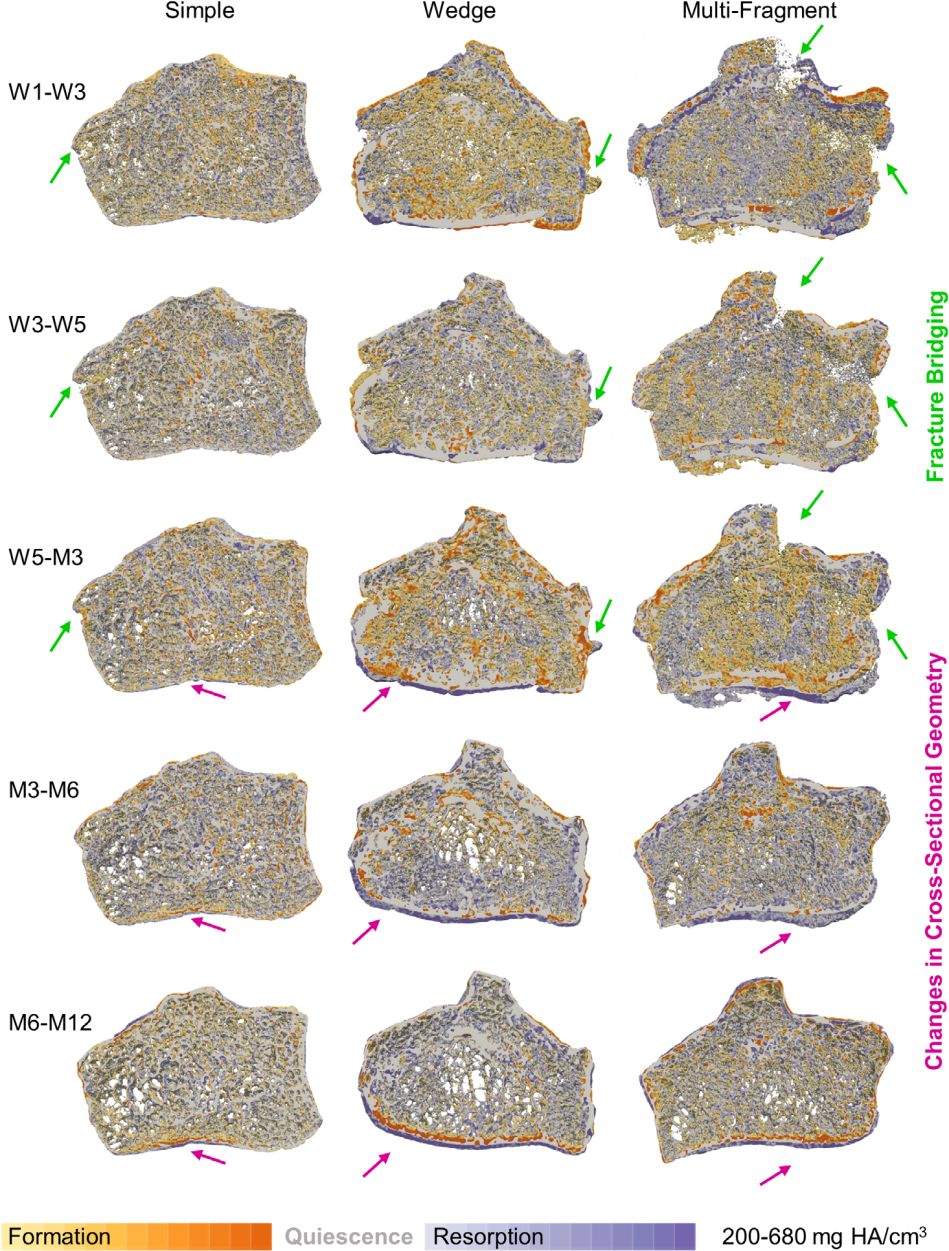
Longitudinal multidensity bone remodeling in a simple (22‐year‐old male; *left*), wedge (52‐year‐old female; *center*), and multifragment (64‐year‐old female; *right*) fracture. Interfragmentary movement is visible between week 1 and week 3 in the multifragment fracture (*top right*). Cortical and trabecular bridging was commonly observed 3‐months postfracture (green arrows placed in the same location within subject). Regions of change in cross‐sectional geometry were observed in mid‐ to late‐stage fracture healing (magenta arrows placed in the same location within subject). HA indicates hyaluronic acid; M, month; W, week.

Visualization of the 3D volumes of formation and resorption shows similar healing trends independent of fracture type and severity (Fig. 6). Specifically, bridging of the fracture was evident at the 3‐month imaging session, independent of fracture severity or cortical gap width; although in the case of a multifragmentary fracture, cortical bridging was more clearly observed at the 6‐month time point. Although subtle in many patients, qualitative morphological differences in cross‐sectional geometry were also observed in the palmar cortex, where the cortex moved inward in late‐stage fracture healing; this was not observed contralaterally.

**Fig 7:**
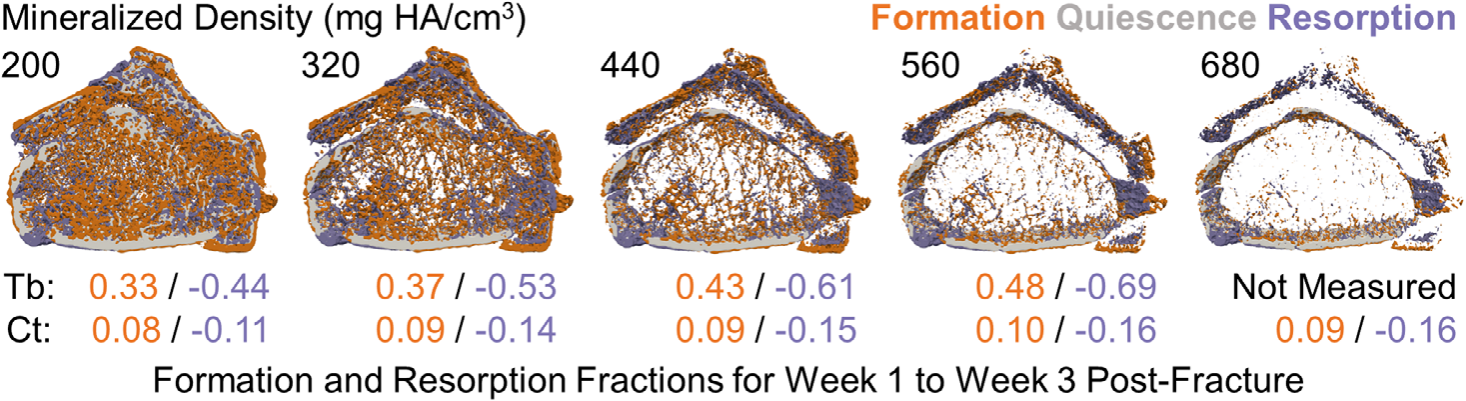
Longitudinal bone formation and resorption fractions between 1‐ and 3‐weeks postfracture are shown using various density thresholds between 200 and 680 mg hydroxyapatite (HA)/cm^3^. The lowest threshold captures large volumes of tissue within the bone, including trabecular (Tb) and cortical (Ct) regions; higher thresholds are isolated to the Ct region.

Trabecular and cortical bone formation and resorption of the fractured radius were higher than in the contralateral radius for all uncasted time points, regions, and densities (Fig. 4), except for the intact regions of W5‐M3 trabecular formation at 200 mg HA/cm^3^, W5‐M3 cortical formation at 920 mg HA/cm^3^, and M6‐M12 cortical resorption at 920 mg HA/cm^3^ (*p* < 0.05 for all other data points). Time frame, bone type, region, threshold, remodeling direction, and the interactions between several of these variables had an effect on formation and resorption volume‐fraction magnitude (Table 4). To further investigate differences in the direction of remodeling, the difference between bone formation and resorption volume fractions was evaluated for each region and density after removal of the cast. No differences were observed in the trabecular fracture nor intact regions. However, in the cortical region, greater resorption was observed at high densities of the fracture region between 5‐weeks and 3‐months postfracture and greater formation was observed for increasing densities with time postfracture in the fracture region and for all densities between 6‐ and 12‐months postfracture in the intact region (Table 6). In culmination, these data indicate that the formation and resorption volume fractions, as well as the ratio between the two, changes not only throughout the duration of fracture healing, but also across densities (Fig. 7).

**Table 6:** Statistically Significant Differences Between Formation (+) and Resorption (−) Volume Fractions for Each Region and Density Threshold of the Fractured Radius

Region		Time postfracture	Density threshold (mg HA/cm^3^)						
			200	320	440	560	680	800	920
Trabecular	Fracture	W5‐M3	–	–	–	–	–		
		M3‐M6	–	–	–	–	–		
		M6‐M12	–	–	–	–	–		
	Intact	W5‐M3	–	–	–	–	–		
		M3‐M6	–	–	–	–	–		
		M6‐M12	–	–	–	–	–		
Cortical	Fracture	W5‐M3	–	0.13 (0.29)	0.15 (0.33)	–	–	–	–
		M3‐M6	–	–	–	0.13 (0.14)	0.15 (0.14)	–	–
		M6‐M12	–	–	–	0.14 (0.14)	0.27 (0.28)	0.39 (0.46)	0.26 (0.31)
	Intact	W5‐M3	–	–	–	–	–	−0.06 (0.12)	−0.11 (0.13)
		M3‐M6	–	–	–	–	–	–	–
		M6‐M12	0.01 (0.02)	0.04 (0.04)	0.05 (0.05)	0.09 (0.06)	0.12 (0.10)	0.12 (0.15)	0.08 (0.07)

Abbreviations: HA, hyaluronic acid. Time postfracture: W1, 1 wk; W3, 3 wk; W5, 5 wk; M3, 3 mo; M6, 6 mo; M12, 12 mo; e.g., W1‐W3 = 1‐wk to 3‐weeks postfracture.

Note: All included values represent statistically significant differences (*p* < 0.05). “–“ represents no difference; empty cells were not analyzed. Differences presented as median (interquartile range).

Other than formation and resorption volume fractions in the adjacent bone regions, the density threshold was a positive predictor of trabecular formation in the intact region (Table 5). Although prediction of resorption in both the fracture and intact regions was generally higher than of formation, the predictability was also higher for trabecular regions compared with cortical regions, which is opposite of what was observed for the contralateral radius (Table 5). No metrics related to patient demographics, densitometric, or morphometric measures; radiographic measurements; prescribed medication; or imaging session were found to be relevant in the prediction of formation and resorption volume fractions in the fractured radius using PLS regression.

## Discussion

Although 3D‐image registration has previously been applied to fractured radii and the combined use of both image registration and bone remodeling analysis has been applied to patients with diminishing bone quality and rheumatoid arthritis,^(^ ^9^, ^10^, ^11^, ^14^ ^)^ our study is the first to evaluate localized bone remodeling throughout the process of fracture healing in humans. Herein, a multidensity approach was used, which parallels recent analysis of bone remodeling in animal fracture healing and allows for differentiation between low‐ and high‐density remodeling.^(^ ^13^ ^)^ By combining the quantification of formation and resorption in the various regions of the fractured radius with 3D visualization of the formation and resorption patterns, we showed that formation and resorption were increased in both the fracture and intact regions of the fractured radius compared with equivalent regions of the contralateral radius, but did not observe any relationships between remodeling and demographics or measures of bone quantity and quality.

Previous studies evaluating fracture healing using longitudinal HRpQCT have generally agreed that BMD increases during the process of fracture healing. However, the specific timeline of this increase is yet to be fully understood. De Jong and colleagues observed increases in trabecular density during the first 6‐ to 8‐weeks postfracture and decreases thereafter with trabecular density at 2 years observed below that measured at 1‐ to 2‐weeks postfracture.^(^ ^7^ ^)^ Conversely, the density of cortical bone decreased through the first 3‐months postfracture, then peaked at 2‐years postfracture. Overall, total bone density increased until 6‐ to 8‐weeks postfracture, then decreased to comparable levels observed initially. Interestingly, Nishino and colleagues investigated BMD directly at the fracture site and observed increases in BMD within the cortical gap and internal callus of the fracture throughout the 6‐month study, whereas increases in BMD of the external callus were slight.^(^ ^15^ ^)^ These findings indicate that although increases in density may occur within the fracture region, the long‐term balance between cortical and trabecular density may be altered postfracture. In agreement with these previous findings, higher density cortical bone formation increased between 3‐months and 1‐year postfracture, which was the latest measurement included herein.

An important consideration for the evaluation of microstructural fracture healing is that of fracture fragment movement, which can result in erroneous/artifactual observations of increased formation and resorption volume fractions. Qualitatively, fracture‐fragment movement was observed predominantly between 1‐week and 3‐weeks postfracture with only subtle fragment motion observed between 3‐ and 5‐weeks postfracture (Fig. 6). Previous research has shown separate registration of each fracture fragment to be a feasible solution to this issue^(^ ^14^ ^)^; however, the fragment‐registration method requires individual segmentation of each fracture fragment and is limited to only sufficiently large fragments. In this previous study, three fragments of an unstable fracture case with clearly visible fragment motion were evaluated; however, only two of these three were able to be registered independently. Thus, segmentation and registration of fragments is only possible for fragments that have sufficient size across stacks and time points, are visually identifiable with a clear fracture gap, and have minimal rotation and translation between imaging sessions. In an effort to maintain as much bone volume in our analysis as possible, we used rigid registration to correct only for motion of the patient between adjacent image‐stack acquisitions; however, future studies should analyze the effect of fragment registration on formation and resorption during fracture healing. Because the registration of fractures with displaced fragments would result in equivalent increases in both bone formation and resorption, it is relevant to note that qualitatively we observed higher resorption than formation in both the fracture and intact regions of the cortical bone between 1 week and 3 weeks and higher formation in the trabecular bone of the fracture region between 3‐ and 5‐weeks postfracture. However, the specific influence of the cast on these observations cannot be isolated. Although the overall process of fracture healing is marked by bone formation during callus mineralization followed by resorption during the remodeling phase, it has been observed at a cellular‐level that there is a peak of nonspecific catabolism (resorption) that occurs before specific anabolism (formation).^(^ ^31^ ^)^ To better understand this mechanism, bone remodeling during early fracture healing should likely be investigated further with smaller time intervals and perhaps alternative imaging modalities that could better capture early‐phase bone healing.

To date, there have only been three studies evaluating longitudinal bone remodeling in humans. The first, from 2014, analyzed 82‐μm voxel HRpQCT images of the radius and tibia in patients with osteoporosis at baseline and 2‐year follow‐up as well as healthy subjects within 1‐month intervals.^(^ ^9^ ^)^ The short‐term images in healthy subjects were used to determine the parameters for analysis with the goal of matching previously observed reproducibility of geometry and density measured using HRpQCT.^(^ ^32^ ^)^ Thereby, a voxel‐by‐voxel subtraction within the largest common volume (periosteal contour) was combined with a difference threshold of 225 mg HA/cm^3^ to determine clusters of formation and resorption, which were considered to be noise and were removed if <30 voxels in size. A follow‐on study by the same research group further analyzed a similar cohort of 82 μm voxel images from postmenopausal women at intervals of 2, 4, and 6 years using the same method; however, here only clusters <five voxels were considered to be noise.^(^ ^10^ ^)^ The most recent study used three combined 10.2‐mm image stacks, acquired with 25% overlap, of 61‐μm voxel images of the second and third metacarpophalangeal joints of patients with rheumatoid arthritis after 6 months.^(^ ^11^ ^)^ This study used within‐acquisition overlap between adjacent image stacks from the same imaging session for ladder‐based registration, then applied a difference threshold of 125 mg HA/cm^3^ and considered clusters of voxels <five voxels in size to be noise. A scan–rescan analysis was then used as a negative control for comparison.

**Fig 8:**
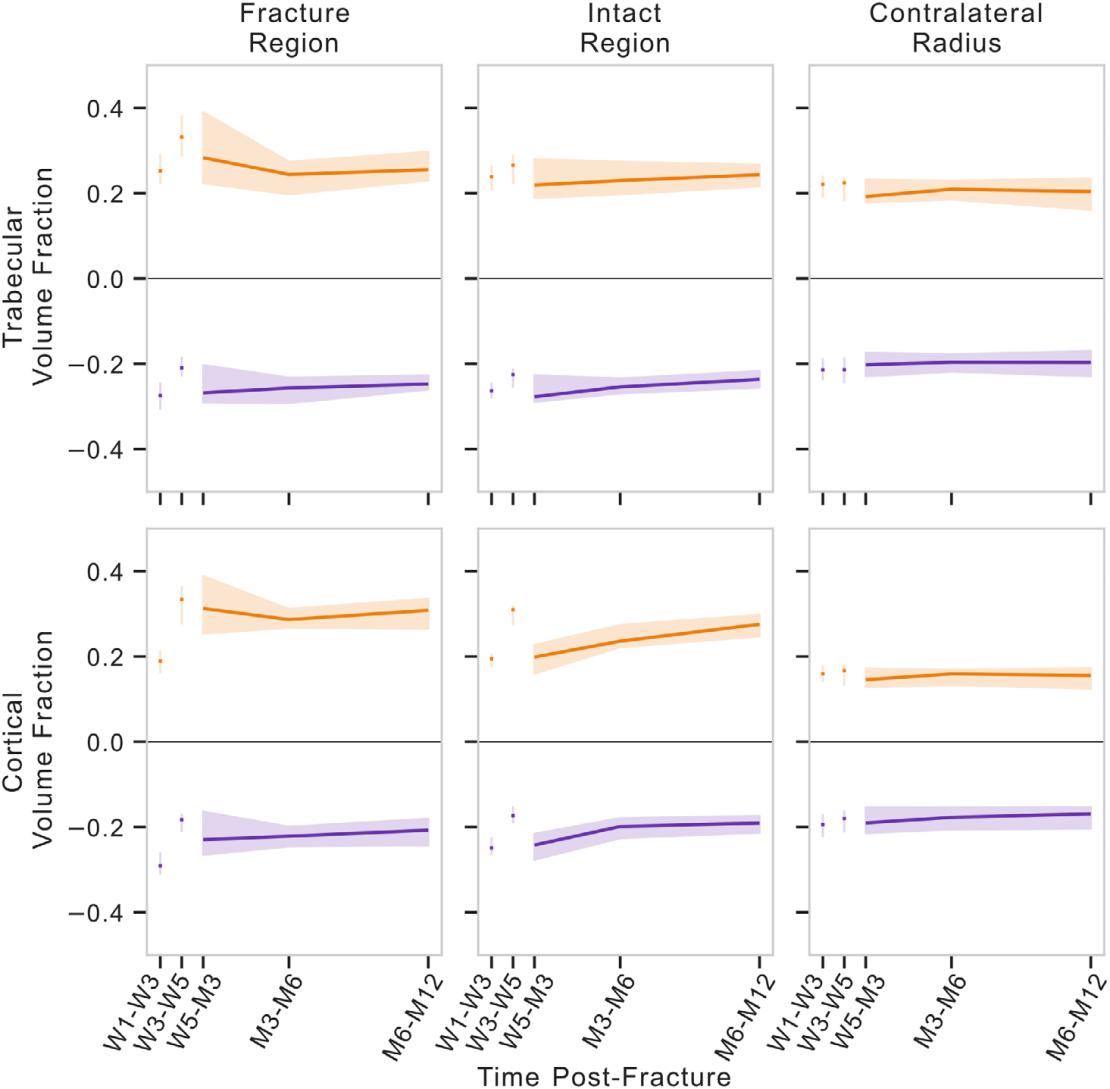
Longitudinal bone remodeling volume fractions for remodeling >125‐mg hydroxyapatite/cm^3^. Plots represent regions of the fractured (*left, center*) and contralateral radii (*right*). Solid line represents median value with a shaded interquartile range. M indicates month; W, week.

Note that the methods in these three studies have used the same overlying concept and methods but have altered thresholds for the size of a cluster that would be considered noise and for the density difference used to identify formation and resorption, without specific justification. Although the formation and resorption volume fractions previously reported have been an order of magnitude lower than those reported herein, there are a few major differences in the analysis that require discussion. Instead of comparing images thresholded to multiple densities, which was done herein and is standard in the analysis of bone remodeling in animals,^(^ ^12^, ^13^ ^)^ these previous studies applied a single, albeit varied, threshold to the difference in density between sequential images. For reference, we have included analysis of our data using the thresholded difference methods of Brunet and colleagues to support the following discussion on methodological differences (Fig. 8).^(^ ^11^ ^)^ Because of the increased density of the cortical bone, the thresholded difference method is much more likely to identify image noise in higher density bone as either formation or resorption, resulting in equivalent or even higher formation and resorption volume fractions in the cortical region than in the trabecular region. This increased formation and resorption in the cortical region can be observed visually in the recent study of bone remodeling in the metacarpophalangeal joint.^(^ ^11^ ^)^ We were specifically interested in investigating fracture healing in both the trabecular and cortical compartments of the distal radius; thus, we chose to instead use a multidensity approach that highlights observed differences at multidensity thresholds. Using this approach, formation and resorption were observed to be lower in the cortical region than the trabecular region, which agrees with our understanding of both fracture healing and bone remodeling, whereby callus mineralization results in low‐density remodeling and trabecular bone turnover is generally greater than that of cortical bone.^(^ ^33^ ^)^ Interestingly, the opposite was observed when the thresholded difference method was applied (Fig. 8).

Another important difference of our method in comparison to previous research is that we did not remove small clusters of voxels in an attempt to remove noise, as this subjective removal of small clusters of formation and resorption may instead ignore subtle changes in bone formation and resorption. The justification for removal of these clusters has largely been a desire to lower observed formation and resorption volume fractions to values of reproducibility of other metrics from HRpQCT and the implementation has been inconsistent, with the size of removed clusters ranging from 1.1 to 16.5 μm^3^ in the only three published studies. Additionally, the removal of small clusters of formation or resorption voxels may inadvertently bias observations of the interplay between cortical and trabecular remodeling caused by differences in morphology and density of the two bone types and our observation that both formation and resorption volume fractions varied with mineralized density.

It is, however, important to note that with our method there is an inherently increased risk of false‐positive identification of formation and resorption, especially at lower density thresholds because of the increased image noise of HRpQCT in comparison with the μCT used in animal studies. Thus, we used the contralateral radius as a reference to identify differences in remodeling associated with fracture healing and avoid any unexpected biasing of the results caused by threshold choice and our lack of noise removal. Although the proportions of density‐based cortical bone volume (Fig. 3) and calculation of BST‐SNR revealed an increased mean density in the contralateral radius in comparison with the fractured radius, the SD of noise was comparable with that of the cast‐free fractured radius (Fig. 2). These differences in density may be a result of varied ROIs during imaging, yet the comparable noise levels and use of volume fractions to quantify remodeling reduce the effects of these differences on the use of the contralateral radius as reference. Even though the time between adjacent imaging sessions varied in length between 2 weeks and 6 months, the different imaging intervals did not result in differences in formation or resorption volume fractions for the contralateral radius. However, we did observe an increase in formation and resorption based on worsening image quality in the contralateral images, with VGS‐1 images having lower formation and resorption volume fractions than other image qualities (Fig. 5). With our use of the contralateral radius of fracture patients as a reference, instead of a separate cohort of patients without a fracture, it is also possible that these data were biased by systemic effects of the fracture, which would be greater during early fracture healing when imaging intervals were the shortest. Although there is a potential that the interplay between imaging interval, systemic effects of fracture healing, and image quality may have disguised more subtle differences in remodeling, the consistency of formation and resorption volume fractions in the contralateral radius indicates that longitudinal studies of 61‐μm HRpQCT bone remodeling may not be appropriate for subjects without disease over time intervals of 6 months or less.

Our patients were treated using a plaster cast, which is the standard‐of‐care at Innsbruck University Hospital. Previous evaluations on the effect of a cast on HRpQCT images found that plaster casts influence bone parameters considerably for both 61‐ and 82‐μm images^(^ ^34^, ^35^ ^)^ through the comparison of densitometrics and morphometrics. In comparison with no cast, plaster casts have been associated with decreases in Tb.vBMD of 4% to 6%, Ct.vBMD of 5% to 7%, and Ct.Th of 2% to 10%. Interestingly, we observed the differences in BST‐SNR with respect to the inclusion of a plaster cast to be isolated to the noise of the soft tissue (Fig. 2). However, we did not evaluate densitometrics and morphometrics because of the lack of validation for the use of these measures on fractured bones or with inclusion of a cast. Although it is understood that beam hardening from a plaster cast results in visually thinner and less‐dense distal cortex and is likely to affect higher densities more than lower densities,^(^ ^34^, ^35^ ^)^ it is still unclear how these factors may have affected the contouring of the periosteum and cortical region in previous studies and what differences changes to the contours may have had on the reported densitometrics and morphometrics. Unfortunately, previous studies have not reported directly on differences in signal or noise; thus, it is difficult to directly compare results or apply any correction to our data. Because images at 1‐week and 3‐weeks postfracture were both acquired with the same cast and images from 5‐weeks postfracture were acquired without a cast, the largest effect of the cast to our results is between the 3‐ to 5‐week images in our study and the reason behind our choice of plotting the first two time points separate from the later time points (Figs. 3 and 4). Even though we did not observe differences in mean signal between images with and without the plaster cast, the removal of the plaster cast before imaging at 5‐weeks postfracture may have affected contouring and region masking, as well as the calculation of bone formation and resorption between 3‐ and 5‐weeks postfracture. This time frame showed indication of greater trabecular formation than resorption in the fracture region and in the highest‐density cortex of both the fracture and intact regions, which may not be independent of cast removal. Thus, these data were not included in any quantitative comparisons. Even though we did not observe an effect of the plaster cast on mean signal of the cortex, future studies should consider treatment using alternative cast materials, such as fiberglass that has been shown to have lesser influence on HRpQCT images, or the inclusion of two scans on the same day before and after cast removal to ensure that the data are consistent across time points and to avoid this potential complication of formation and resorption calculations.

Cortical bone formation volume fractions were significantly greater than resorption volume fractions with increasing density and time postfracture in the fracture region and at all densities of late‐stage fracture healing in the intact region (Table 6); however, both formation and resorption were generally higher in all four compartments (bone type and region) of the fractured radius in comparison with the contralateral radius throughout the duration of the study. Additionally, we observed large changes in morphology between 6‐ and 12‐months postfracture (Fig. 6), indicating that the remodeling phase of fracture healing continues through this time frame. This late‐stage morphological remodeling could be a result of altered loading based on imperfect fracture reduction and should be investigated in the future by combining morphological and mechanical analyses. Previous studies have reported a restoration of morphometrics after 2‐years postfracture^(^ ^7^ ^)^; therefore, future longitudinal studies should consider extending the analysis period beyond the first‐year postfracture.

Although previous research has found a number of factors (e.g., age, radiographic measures, dominance of the fractured side, etc.) to influence patient outcome postdistal radius fracture,^(^ ^36^ ^)^ other aspects, such as injury characteristics, which would seem important to patient outcome, have been found to be irrelevant to patient outcomes.^(^ ^37^ ^)^ Although our investigation did not find any clear relationships of bone formation and resorption with other clinical factors, the quantification of bone remodeling indicated a dependence on mineralized bone density (Tables 4 and 5) that is relevant to our understanding and future investigation of distal radius fracture healing. Although bone resorption of the fractured radius was relatively consistent across mineralized density thresholds, formation showed larger variation at higher thresholds than lower thresholds over the course of the study, especially in the cortical bone of the fracture region. We observed peak formation for nearly all densities of the intact and fractured regions of the fractured radius between 3‐ and 5‐weeks postfracture after an initial peak of resorption that occurred between 1‐week and 3‐weeks postfracture. Nevertheless, it must be considered that these observations may be artifactual because of the inclusion of the cast in the 1‐ and 3‐week images. The observation that formation is density‐dependent and occurs in both the fractured and intact regions of the cortex during late‐stage fracture healing may be relevant to fracture immobilization strategies, especially for patients with compromised bone quantity and quality. Importantly, our study also highlighted subtle aspects of fracture healing, including the progression of cortical bridging and late‐stage changes in cross‐sectional geometry, which deserve more in‐depth analysis and quantification. Specifically, future research should incorporate methods to more thoroughly investigate both morphology through parameterization^(^ ^38^ ^)^ or direct shape analysis and mechanically driven remodeling through finite element analysis and mechanoregulation^(^ ^13^ ^)^ to further our understanding of how clinical treatment strategies, such as fracture reduction or surgical intervention, may influence fracture healing in patients with varying bone quantity and quality.

## Author Contributions

**Penny Atkins:** Data curation; formal analysis; investigation; methodology; software; visualization; writing‐original draft; writing‐review & editing. **Kerstin Stock:** Data curation; resources; writing‐review & editing. **Nicholas Ohs:** Methodology; software; writing‐review & editing. **Caitlyn Collins:** Methodology; writing‐review & editing. **Lukas Horling:** Data curation; resources. **Stefan Benedikt:** Data curation; resources. **Gerald Degenhart:** Data curation; writing‐review & editing. **Kurt Lippuner:** Funding acquisition; writing‐review & editing. **Michael Blauth:** Conceptualization; funding acquisition. **Patrik Christen:** Conceptualization; funding acquisition. **Ralph Mueller:** Conceptualization; funding acquisition; writing‐review & editing.

### Peer Review

The peer review history for this article is available at https://publons.com/publon/10.1002/jbm4.10493.

## Supporting information


**Appendix S1**: Supporting information
