# Supplementary material for: Formation Dominates Resorption With Increasing Mineralized Density and Time Postfracture in Cortical but Not Trabecular Bone: A Longitudinal HRpQCT Imaging Study in the Distal Radius
Source: JBMR Plus. 2021 Apr 8;5(6):e10493. doi: 10.1002/jbm4.10493 (PMC8216136; doi:10.1002/jbm4.10493)
Supplement: Supplementary file 1 — Appendix S1: Supporting information [file JBM4-5-e10493-s001.docx]

Image Registration

HR-pQCT images were cropped in the transverse plane to the region surrounding the radius and ulna. A dilated mask, which excluded the ulna, was generated to isolate the radius and surrounding soft tissue prior to image registration. Image registration was performed in Python (v3.7)^(1)^ using a previously described pyramid-based approach.^(2)^ Herein, images were down-sampled to one-sixteenth of the original resolution (971.2 µm voxels) and were coarsely registered. The down-sampled registration parameters, including translations and rotations in three-dimensions, were then transformed and used as initial registration parameters for the original images down-sampled to a finer resolution (i.e. one-eighth the original resolution or 485.6 µm voxels). This process was repeated iteratively with the final registration optimization on the original resolution images (60.7 µm voxels). Registration was optimized relative to the mean squared error between two images and was confirmed visually in all cases to ensure correct registration.

For registration of the standard image stack, the stack from the first appointment was rotated about the long axis of the bone to visually align with the image coordinate system and transformed using cubic interpolation using the SciPy function library.^(3)^ The transformed stack was then used as a target for registration of the stack from the second imaging session. Stacks from the following imaging sessions were then iteratively registered and transformed using cubic interpolation to the stack acquired at the previous imaging session to reduce any effect of long-term morphological changes on image registration. For both the contralateral and fractured registered images, the proximal and distal axial slices which were not fully visible from all six imaging sessions were removed from further analysis.

For the three stacks of the fractured radius, the standard image stack registration process was first applied to the middle stack of all timepoints. While the three stacks were acquired one after another, the image stacks were often not contiguous due to subtle patient motion within or between each image stack acquisition. For this reason, proximal and distal image stacks of the fractured radius were registered independently using an iterative ladder-based image registration approach, similar to the process used by Brunet et al.,^(4)^ except the overlap between image stacks herein was between timepoints due to inaccuracies in placement of the reference line instead of within a timepoint due to overlap between image stacks (Figure 1). Thus, the full set of registered middle stack images for each patient were assessed to identify the image pair for each subject that had sufficient overlap and would be used for the ladder-based registration approach of the proximal and distal image stacks. In general, image pairs were chosen such that the middle stacks were the most distally- and proximally-located middle stacks (Figure 1; teal and gold radii, respectively); however, the choice also considered image quality and proximity in time, with a preference for higher quality and later images to minimize the changes due to fracture fragment movement or callus deposition and resorption. The distally-located middle stack was then used as the target for registration of the proximally-located distal stack and the proximally-located middle stack as the target for registration of the distally-located proximal stack. These registered proximal and distal image stacks were then used as the target registered images for the proximal and distal stacks, respectively. The remaining proximal and distal image stacks were then iteratively registered to images from adjacent imaging sessions until all three image stacks of each image set were registered. For each imaging appointment, the three image stacks were combined into a single image volume, where the maximum density value was assigned for any voxels that overlapped between two images and a longitudinally-calculated interpolated value was assigned for any voxels that were missing between the two image stacks.

While the pyramid-based registration algorithm was able to correct for smaller differences in image position, there were often large offsets and rotations between imaging sessions, thus initial parameters for rotation about and translation along the long-axis of the bone were provided to achieve acceptable convergence of the algorithm. For registration of the proximal and distal image stacks, the registration parameters of the middle stack were used as initial parameters. When only a small amount of overlap existed between image stacks of adjacent timepoints (i.e. during ladder-based image registration), there were limited features for registration, or the fracture included significant callus or fragment motion, a set of manually derived parameters for the rotation angles and translations was visually determined and used as input to the registration (42% of distal stacks, 48% of standard stacks, and 61% of proximal stacks for the fracture images; 17% of standard stacks for the contralateral images). In the case of minimal overlap between the image stacks being registered, the down-sampling of the pyramid approach often reduced the overlap between images such that they could not be registered; thus, image down-sampling was then limited to one iteration (i.e. half resolution).

Region Masking

As there is no standard protocol for the analysis of fractured radii, contours of the radius, as well as specific regions of the contoured radius were generated using custom scripts in Python. The outer contours of the radii were generated for each image using an iterative geodesic active contouring method developed by Ohs and colleagues^(5)^ with manual edits performed in the scanner manufacturer’s graphical user interface, as necessary, to include localized segments of thin distal cortical bone, which had high curvature and did not differentiate well from the background. From this outer contour, cortical and trabecular masks were generated using a custom approach to provide a consistent method to isolate the cortical and trabecular regions of the fractured radii. Herein, the voxels outside of the radius contour were set to 360 mg HA/cm^3^ to promote inclusion of thin and less dense distal cortex in the mask after the application of a constrained Gaussian filter (sigma=4, truncate=4, support=16, where the support is computed by the library through multiplication of the truncate and sigma parameters) using the Python SciPy function library.^(3)^ The largest connected component of voxels with density greater than 360 mg HA/cm^3^ was then assigned to the cortical region, which helped to remove regions of denser and thicker trabecular bone, but unfortunately also excluded a rare highly dislocated fragment that was not in contact with any bone surface. Importantly, the identified cortical region did not include a consistent cortical wall due to the thin and less dense distal cortex (Figure 1); however, this was preferred over the creation of an artificially connected cortex, which would bridge fracture fragment gaps. The trabecular region was defined as all voxels within the outer contour of the radius that were not assigned to the cortical region. The cortical and trabecular regions of the contralateral radius were defined using the standard process implemented in the manufacturer’s software, which does not require user-defined parameters.

Following segmentation of the cortical and trabecular regions, the fracture region was then manually identified using 3D surface reconstructions, which were generated using a threshold of 320 mg HA/cm^3^ (ParaView, Kitware Inc., v5.4.1)^(6)^, and visualization of the axial HR-pQCT images. Points along the visible fracture region were selected using the images three-weeks post-fracture to reduce error due to fracture fragment movement, which occurred predominantly between one- and three-weeks post-fracture, and to minimize the influence of callus mineralization, which was clearly visible by five-weeks post-fracture, on region selection. Selected points were dilated by 30 voxels using a distance transform and Gaussian filtered (sigma=20, truncate=4, support=80) prior to the application of a threshold at 50% (Python SciPy function library^(3)^, Figure 1). The resultant fracture region mask was visually verified to ensure all identified fracture regions were included in the mask. When sections of the fracture did not appear to be fully covered by the mask, additional points were selected, and the fracture mask was regenerated. The fracture mask was then used for the images of all six time points.

References

1. Python Software Foundation. Python Language Reference, Version 3.7 [Internet]. 2020. Available from: https://www.python.org

2. Thévenaz P, Ruttimann UE, Unser M. A pyramid approach to subpixel registration based on intensity. IEEE Trans. Image Process. IEEE Trans Image Process; 1998;7(1):27–41.

3. Virtanen P, Gommers R, Oliphant TE, Haberland M, Reddy T, Cournapeau D, Burovski E, Peterson P, Weckesser W, Bright J, van der Walt SJ, Brett M, Wilson J, Millman KJ, Mayorov N, Nelson ARJ, Jones E, Kern R, Larson E, Carey C, Polat İ, Feng Y, Moore EW, VanderPlas J, Laxalde D, Perktold J, Cimrman R, Henriksen I, Quintero EA, Harris CR, Archibald AM, Ribeiro AH, Pedregosa F, van Mulbregt P, Contributors S 1. 0. SciPy 1.0--Fundamental Algorithms for Scientific Computing in Python. 2019;1–22. Available from: http://arxiv.org/abs/1907.10121

4. Brunet SC, Kuczynski MT, Bhatla JL, Lemay S, Pauchard Y, Salat P, Barnabe C, Manske SL. The utility of multi-stack alignment and 3D longitudinal image registration to assess bone remodeling in rheumatoid arthritis patients from second generation HR-pQCT scans. BMC Med. Imaging. BioMed Central Ltd.; 2020 Apr 7;20(1).

5. Ohs N, Tourolle DC, Atkins PR, Collins CJ, Schroeder B, Blauth M, Christen P, Müller R. Automated Segmentation of Fractured Distal Radii by 3D Geodesic Active Contouring of in vivo HR-pQCT Images. bioRxiv [Internet]. Cold Spring Harbor Laboratory; 2020 Oct 14 [cited 2020 Oct 15];2020.10.14.339739. Available from: https://doi.org/10.1101/2020.10.14.339739

6. Ayachit U. The ParaView Guide: A Parallel Visualization Application. Kitware; 2015.
